# Epigenetic Targets and Pathways Linked to SARS-CoV-2 Infection and Pathology

**DOI:** 10.3390/microorganisms11020341

**Published:** 2023-01-30

**Authors:** Ali A. Rabaan, Mohammed Aljeldah, Basim R. Al Shammari, Roua A. Alsubki, Jawaher Alotaibi, Yousef N. Alhashem, Neda A. Alali, Tarek Sulaiman, Zainab Alsalem, Huda A. Bajunaid, Mohammed Garout, Heba A. Alsaffar, Souad A. Almuthree, Doha Hudhaiah, Azhar M. Alzaher, Fatimah A. Alshaikh, Amer Alshengeti, Mustafa A. Najim, Ramadan Abdelmoez Farahat, Ranjan K. Mohapatra

**Affiliations:** 1 Molecular Diagnostic Laboratory, Johns Hopkins Aramco Healthcare, Dhahran 31311, Saudi Arabia; 2 College of Medicine, Alfaisal University, Riyadh 11533, Saudi Arabia; 3 Department of Public Health and Nutrition, The University of Haripur, Haripur 22610, Pakistan; 4 Department of Clinical Laboratory Sciences, College of Applied Medical Sciences, University of Hafr Al Batin, Hafr Al Batin 39831, Saudi Arabia; 5 Department of Clinical Laboratory Sciences, College of Applied Medical Sciences, King Saud University, Riyadh 11362, Saudi Arabia; 6 Infectious Diseases Unit, Department of Medicine, King Faisal Specialist Hospital and Research Center, Riyadh 11564, Saudi Arabia; 7 Clinical Laboratory Science Department, Mohammed Al-Mana College for Medical Sciences, Dammam 34222, Saudi Arabia; 8 Pediatric Department, Security Force Hospital, Riyadh 13526, Saudi Arabia; 9 Infectious Diseases Section, Medical Specialties Department, King Fahad Medical City, Riyadh 12231, Saudi Arabia; 10 Department of Epidemic Diseases Research, Institute for Research and Medical Consultations (IRMC), Imam Abdulrahman Bin Faisal University, Dammam 31441, Saudi Arabia; 11 Makkah Specialized Laboratory, Fakeeh Care Group, Hadda 25321, Saudi Arabia; 12 Department of Community Medicine and Health Care for Pilgrims, Faculty of Medicine, Umm Al-Qura University, Makkah 21955, Saudi Arabia; 13 Department of Azizia Primary Health Care, Ministry of Health, Dammam 32211, Saudi Arabia; 14 Department of Infectious Disease, King Abdullah Medical City, Makkah 43442, Saudi Arabia; 15 Microbiology Laboratory, King Fahad University Hospital, Al Khobar 34445, Saudi Arabia; 16 Improvement of Operation Department, Qatif Health Network, Qatif 31911, Saudi Arabia; 17 Infection Prevention and Control Department, Qatif Central Hospital, Qatif 32654, Saudi Arabia; 18 Department of Pediatrics, College of Medicine, Taibah University, Al-Madinah 41491, Saudi Arabia; 19 Department of Infection Prevention and Control, Prince Mohammad Bin Abdulaziz Hospital, National Guard Health Affairs, Al-Madinah 41491, Saudi Arabia; 20 Department of Medical Laboratories Technology, College of Applied Medical Sciences, Taibah University, Madinah 41411, Saudi Arabia; 21 Faculty of Medicine, Kafrelsheikh University, Kafrelsheikh 33511, Egypt; 22 Department of Chemistry, Government College of Engineering, Keonjhar 758002, India

**Keywords:** SARS-CoV-2, COVID-19, epigenetics, drug targets, COVID-pathology

## Abstract

The scale at which the SARS-CoV-2/COVID-19 pandemic has spread remains enormous. Provided the genetic makeup of the virus and humans is readily available, the quest for knowing the mechanism and epidemiology continues to prevail across the entire scientific community. Several aspects, including immunology, molecular biology, and host-pathogen interaction, are continuously being dug into for preparing the human race for future pandemics. The exact reasons for vast differences in symptoms, pathophysiological implications of COVID-infections, and mortality differences remain elusive. Hence, researchers are also looking beyond traditional genomics, proteomics, and transcriptomics approach, especially entrusting the environmental regulation of the genetic landscape of COVID–human interactions. In line with these questions lies a critical process called epigenetics. The epigenetic perturbations in both host and parasites are a matter of great interest to unravel the disparities in COVID-19 mortalities and pathology. This review provides a deeper insight into current research on the epigenetic landscape of SARS-CoV-2 infection in humans and potential targets for augmenting the ongoing investigation. It also explores the potential targets, pathways, and networks associated with the epigenetic regulation of processes involved in SARS-CoV-2 pathology.

## 1. Introduction

COVID-19 has taken millions of lives and affected almost the entire human population socio-economically [1,2]. The disease is reportedly known to have originated in Wuhan City of Hubei Province in China. It was caused by a new strain of a previously known group of viruses called novel coronavirus or SARS-CoV-2, which can spread via contact or aerosol and remains highly sporadic [3]. According to the World Health Organization the first recorded case was observed in December 2019 in Wuhan [2]. Through samples analysed by metagenomic RNA-sequencing of BAL fluid viral isolates of patients with symptoms of severe pneumonia, independent research groups in China identified the causative agent as a betacoronavirus for the first time, which was never detected earlier. Finally, on 9 January 2020, the identification was publicly announced and witnessed worldwide [4,5]. On 10th January 2020 the genomic sequence of the coronavirus was published for the first time on the virological website [4,5,6,7]. Later, a few more patients were discovered with no previous contact record with Huanan Seafood Wholesale Market [8]. The study provided concrete evidence that this new virus can be spread from person to person. Migration between cities also increased the rate of spread in China. Because the epidemic occurred just before the Chinese lunar New Year it quickly spread to other regions of the country [9], and the virus had already spread to the 34 Chinese provinces in just one month [5]. The number of confirmed cases began to increase dramatically, with an increase of more than thousands each day in January 2020. The novel coronavirus infection was classified as a global epidemic by the WHO on January 30 [2]. On February 11 the WHO and the International Committee on Taxonomy of Viruses gave the infection the designation COVID-19. However, international dissemination of COVID-19 increased from late February despite the downward trend in China [2]. The global COVID-19 spread was formally classified as a pandemic by the WHO on March 11, 2020. Globally, more than 30 million cases were identified over the next ten months. A total of 526,558,033 confirmed cases of COVID-19, including 6,287,117 fatalities, were reported to WHO as of the time this book was written, and 11,811,627,599 doses of vaccine have been given as of 23 May 2022. According to the WHO there have been 524,630 deaths and 43,158,087 confirmed cases of COVID-19 in India [2]. In addition, a total of 1,941,328,608 vaccine doses have been administered. SARS-CoV-2 is an endogenous virus that resides in animals, according to genetic evidence, but it is still unknown when and from where the virus first infected humans. It was suggested that the seafood market may not have been the primary site of human infection with SARS-CoV-2, as some of the first cases in Wuhan had no epidemiological connection to the above-mentioned region [8]. However, this was debated and did not rule out the possibility of false positives and contaminations. At the same time, the origin of the virus was investigated in light of previous cases of Nipah and Hendra, which were bat-borne [10]. Such a virus transfers from one organism to humans is called zoonotic transfer and the pathogen is called zoonotic [11]. The exact zoonotic cascade of SARS-CoV-2 has yet to be completely understood. Previously, in 2005, horseshoe bats are known to be natural hosts of coronaviruses and therefore speculated to be responsible for the current SARS-CoV-2 [12]. COVID-19 spreads through fomites or aerosoles, making it unsafe to touch the infected individual. Furthermore, the faecal–oral route is not yet an absolutely proven route and needs to be further explored for transmission relevance [13]. Humans typically contract minor to severe respiratory diseases due to coronavirus infection. The CDC reports that patients with COVID-19 infection displayed a wide variety of symptoms, from little discomfort to serious sickness. As soon as 2 days or within 14 days after virus exposure, symptoms may start to show [14]. Breathing difficulties, fever with or without chills, frequent dry cough, exhaustion, muscular or body soreness, headache, lack of taste or smell, congestion or a runny nose, nausea or vomiting, and occasionally diarrhoea are some of the symptoms that are frequently observed [15]. As per the regulatory bodies such as centre for disease control (CDC) this list may not be exhaustive, and some additional symptoms may also appear. Severe complications from COVID-19 infection are seen primarily in older adults and those with significant concomitant medical disorders, including diabetes and serious heart or respiratory-related illnesses [14].

### 1.1. SARS-CoV-2 Structure

**Figure 1:**
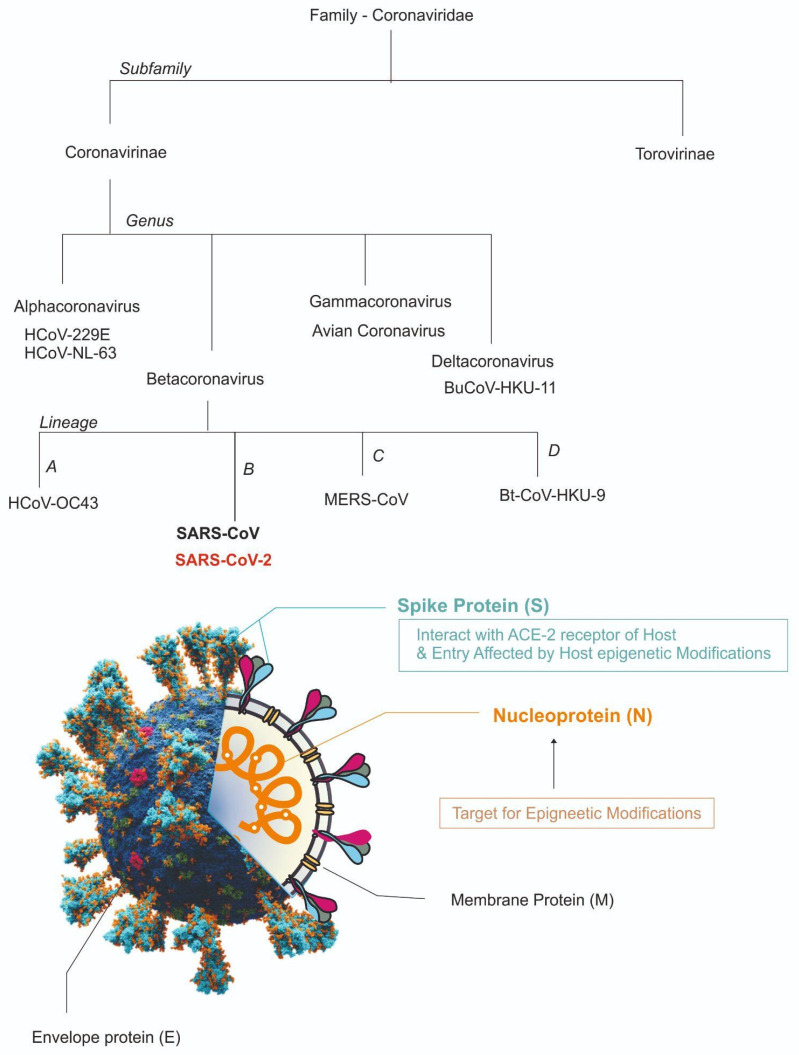
Overview of the classification of SARS-CoV-2 and its structure [ACE2, angiotensin I converting enzyme 2; E, envelope; M, membrane; N, nucleocapsid; RNA, ribonucleic acid; S, spike; SARS-CoV-2, coronavirus 2 of severe acute respiratory syndrome coronavirus 2].

SARS-CoV-2 structure, or similar viruses, can be studied by observing its two major components, the outer protein envelope made up of envelope proteins, and inner core made up of the nucleoprotein complex and its genome. SARS-CoV-2 is a relatively larger virion having an average diameter of 60–140 nanometres. The S (spike), E (envelope), M (membrane), and N (nucleocapsid) proteins are the four structural proteins that make up SARS-CoV-2, as with other coronaviruses. The N protein protects the viral RNA genome, and the S, E, and M proteins combinedly work to develop the viral envelope [16]. Glycoproteins and type I membrane proteins known as spike proteins (S) have a single transmembrane domain oriented to the membrane’s extracellular face. S1, or the stalk fusion domain, and S2, or the globular receptor domain, are the two functional components that make up spike proteins [17]. The S1 subunit catalyzes the attachment of this protein to the host receptor, while the S2 subunit catalyses fusion [18]. This spike protein, known to interact with human angiotensin-converting enzyme (ACE) proteins, enables the virus to adhere to and fuse with the membrane of a host cell. The spike proteins are also the first proteins which elicit the neutralising antibody formation in the host [19]. The virus develops a special phenotypic that produces spikeless, non-infectious virions that contain M but no S protein when tunicamycin is present [20]. Additional structural and accessory proteins, for example, the HE protein, the 3a/b protein, and the 4a/b protein, which are used primarily during genomic maintenance and virus replication, are found in different CoV, in addition to the primary structural proteins [21]. Coronaviruses are equipped with one of the largest genomes of RNA viruses, with 26.4 to 31.7 kb of genetic material [22]. The SARS-CoV-2 genome is a linear, positive-sense, the single-stranded RNA genome of 29,881 bp in length (GenBank no. MN908947), encoding 9860 amino acids. The proportion of U in the viral genome is the highest (32.2%), followed by that of A (29.9%), and G and C have equal proportions (19.6% and 18.3%, respectively). Like other coronaviruses, its genome predisposes against nucleotides cytosine (C) and guanine (G) [23]. Genetic fragments produce both structural and non-structural proteins. S, E, M, and N genes produce structural proteins, whereas the ORF region encodes nonstructural proteins such as 3-chymotrypsin-like protease, papain-like protease, and RNA-dependent RNA polymerase [24]. In the clinical diagnosis of COVID-19 via PCR, Orfs are quite helpful [25]. As S, E, M, and N5A typical CoV comprises at least six ORFs in its genome, all coronaviruses’ key structural protein genes occur in the order 5′-3′ as S, E, M, and N. The structure and outline classification of the SARS-CoV-2 is illustrated in Figure 1.

### 1.2. Entry Mechanism of Virus in Host Cell and Replication

The glycosylated spike protein on the surface of SARS-CoV-2, which is known to bind with angiotensin-converting enzyme 2 (ACE2) on the surface of host cells, facilitates the virus’ entrance [26]. Activating the bound spike protein by the 2TM type proteases on the host cell surface, specifically the TM protease serine 2 (TMPRSS2), makes entry into the host cell easier [27]. All human coronaviruses (HCoVs) share the SARS-CoV-2 S protein, which is highly conserved and involved in receptor recognition, viral attachment, and penetration into host cells [28]. Upon entering the cell, the virus releases its genetic material, follow by replication and transcription, and then the replicase-transcriptase complex is assembled.

The initial stage of the life cycle in host cells involves the virus spike protein interacting with host cells through the host’s ACE2 receptors of the host (along with other co-receptors) to internalize host cells [29]. The virus is internalised through clathrin pits and endocytosis thereafter. When RNA is released it exploits the cytoplasm of the host cell for simultaneous transcription, translation, vacuole formation, and budding. The cell eventually exocytosis the freshly generated virus particles and releases them [30].

## 2. General Context of Epigenetics

Epigenetics is the study of changes in genetic outcomes imparted by behaviour and environment. While genetic changes pertain to mutations in the basic alphabet of information mostly remain unidirectional and hence irreversible, the epigenetic changes are readily reversible and do not change the basic genetic alphabet, but the pattern of reading the alphabet. Currently, a plethora of diseases, behaviours, and various health indicators have been associated with one or more epigenetic mechanisms [31]. Diseases such as cancers, cognitive dysfunction, and respiratory, metabolic syndromes, cardiovascular and renal diseases, autoimmune diseases or neuro-cognitive illness are mostly known to be linked with epigenetic regulation [32]. In its literal sense, the term “epigenetic” means “in addition to alterations in genetic sequence.” With newer studies, this phrase has expanded to include several events that leads to gene modification without the alteration of DNA sequence and result in modifications which, if some of these modifications could be undone, may be passed on to daughter cells. Epigenetics is described as “the study of mitotically or occasionally meiotically heritable changes in gene expression that are not induced by changes in DNA sequence” in yet another description provided by Waterland [33]. On the other hand, much broader definitions provided by heath regulatory bodies depict epigenetics as a broader domain, which may not essentially encompass the criterion of inheritance. In their recent epigenomics programme, the US National Institutes of Health suggested that epigenetics might involve both inherited genetic changes in gene activity and non-heritable long-term variations in the transcriptional potential of a cell. However, clear mechanisms and definitions continue to be debated and remain unless a deeper insight is achieved [34]. The most prevalent epigenetic mechanisms are methylation (-CH3 addition), acetylation (-COCH3 addition), phosphorylation (-PO4 addition), ubiquitylation (-Ubq addition), and sumolyation (-Small Ubiquitin-like Modifier protein addition). Aforementioned post-translational modification, particularly those of histone proteins, manifest as remodelling of chromatin and RNA-based mechanisms to cause their effects in an organism [34]. Although it is known that epigenetic processes happen naturally and are essential to many aspects of organismal function, when they go wrong they can have serious negative impacts on both health and behaviour [32]. Perhaps the most well-known epigenetic activity is cytosine methylation, or the insertion of a methyl group (CH3), most frequently where cytosine bases occur consecutively. This may be because it is simple to investigate with current equipment. Since its discovery in many other diseases and health situations in 1983, DNA methylation has been linked to numerous illnesses and health issues, including human cancer [35,36]. There are thousands of gene-activities that are regulated by epigenetic modifications and therefore affect the phenotype. Epigenetic modifications not only regulate the start or end of a transcription process, but also affect the pace and integrity of the transcriptional status. Epigenetic changes also specify the manner in which any gene responds to external stimuli [37]. In mammalian DNA most methylated cytosine is present in 5′-CpG-3′ dinucleotides often called CpG islands [35]. The frequency of methylation in non-CpG sequences such as 5′-CpNpG-3′, nonsymmetrical 5′-CpA-3 ′ and 5′-CpT-3′ is often substantially lower. In addition to the essentially distinct and independent modifications some histone changes depend on each other, and given the right circumstances can occur simultaneously at the same genomic loci [38]. This makes epigenetic understanding even more difficult. For instance, histone lysine residues can be monomethylated, dimethylated, or trimethylated, but arginine residues can be monomethylated, symmetrically, or asymmetrically dimethylated, with each change having a particular physiological consequence [39]. Therefore, researchers refer to the combination of covalent changes that occur in conjunction with DNA methylation, such as alteration in the chromatin structure and change in gene’s transcription as “histone code.” [40]. This histone code is then “read” by transcriptional modules present in the chromatin regulators such as bromodomain, chromodomain, and Tudor domain causing the ultimate effect of epigenetic changes [41]. The chromatin network made of histones protein complex and DNA is tightly bound to fit inside the nucleus. These complexes result in the formation of either euchromatin (transcriptionally active chromatin) or heterochromatin (partially inactive chromatin), which can be altered by compounds like acetyl groups (acetylation), enzymes, and various types of RNA including microRNA0s and small interfering RNAs [42]. In order to control the gene expression from a particular set of chromatin networks in an organism, epigenetic factors are frequently involved in the remodelling of chromatin. The inheritance of epigenetic changes is still debatable and there is little evidence on mechanisms, although the phenomenon has been observed in several model organisms and humans [43]. In some cases, the passing down of epigenetic changes can have a significant impact on the progeny and theoretically offer an adaptive advantage [44,45]. However, epigenetic transmission may not always be beneficial, but a load built over a lifetime may potentially bring disease risk factors or their predisposition [46]. Although some deeper aspects of epigenetics have yet to be unravelled, the development of drugs and therapies based on epigenetic modification has already begun, which has attracted both researchers and the pharma sector.

## 3. Tools for the Study of Epigenetics

**Figure 2:**
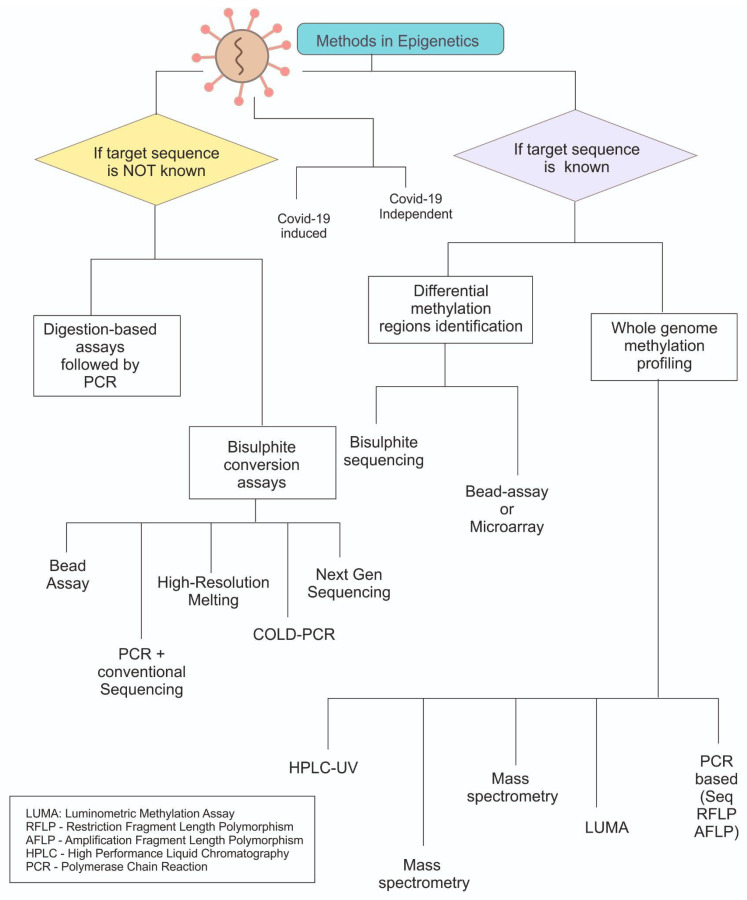
Overview of the methods used for epigenetic analysis. Primarily categorised based on knowledge about the gene sequence and later on the approaches. Most methods which are based on bisulphite need to derivatize the modifications before they can be analysed.

Since epigenetics was coined in 1940, the advent of epigenetic studies gained momentum and researchers, and within the next two decades DNA methylation and other histone modifications were elucidated to a great extent. In subjective terms, methods for the study of epigenetics can be categorised into two different genres, one classical and one modern. The classical methods to study epigenetics involve bisulfite sequencing, PCR, mass spectrometry and associated techniques described in suitable context below. Figure 2 summarises the common methods and decision making for a choice.

### 3.1. Bisulfite Sequencing

Bisulfite sequencing involves the treatment of bisulfite reagent followed by sequencing. As methylation commonly occurs at cytosine residue transforming the residue to 5’ methylcytosine. During the bisulphite treatment unmethylated cytosine modified into uracil by the methylated cytosine does not change, which helps in the identification of modified sites by sequencing technique [47]. The allelic changes after the denaturation are identified using PCR methods that are specific for methylation and non-methylation identification [48]. Bisulfite sequencing has recently been used for finding the DNA methylation pattern in COVID-19 patients. A preprint article by Winky et al. identified that virus-specific differentially methylated regions (vDMR), and stated that these regions are responsible for the weakest vs. strongest epigenome responses. One of the noteworthy results was a considerable replication rate of 42% in SARS-CoV-2 vDMR that was seen in separate samples, which demonstrated severe epigenome alteration upon infection [49].

### 3.2. Non Methylation Specific PCR Procedures

Besides conventional sequencing, some PCR-based methods involving the epigenetic modifications have also been developed, which are mainly based on nonmethylation specific PCR, yet identification of conventional polymorphism in the genetic material. Amplified fragment length polymorphism (AFLP) [50] and restriction fragment length polymorphism (RFLP) are examples of PCR variants that are often used [51]. Procedures often involve fractionating the sequences and then extracting them from the polyacrylamide gel to isolate differentially methylated sequences. Although these methods are inexpensive, they are becoming obsolete and less frequently used as new methods have now emerged such as next generation sequencing, global methylation, etc.

### 3.3. DNA Sequencing and Microarray

The most common and frequently used technique for assessing epigenetic alteration in nucleic acids is DNA sequencing, particularly Next Generation Sequencing (NGS) analysis [52]. In general, bisulfite specific primer sequences are employed for distinction of the sites. The unmethylated cytosines change into thymine in the resultant amplified sequences. Adenines serve as the equivalent residues on the antisense strand [53]. Among various NGS methods, illumina platforms are widely used and remain most successful for the analysis. The method compares the number of individual residues ingested when the sequence to determine the ratio of the C:T residues. In addition to DNA sequencing, oligonucleotide microarrays designed for CpG sites are also an alternative choice for the identification of nucleic acid epigenetic modification, allowing genome-wide data on methylation although mainly used for polymorphism identification [54].

### 3.4. Methylation-Sensitive Single-Nucleotide Primer Extension (MS-SnuPE)

This technique is mainly used for the quantification of methylated DNA. This helps in the identification of methylated CpG sites using multiple oligonucleotides by utilizing the single primer extension reaction. In this method, the DNA is initially treated with sodium bisulfite and purified. This is followed by the amplification of treated DNA using PCR and then the PCR product is isolated with the help of agarose gel electrophoresis technique [55]. The isolated PCR product is then annealed with MS-SnuPE primer and then single-nucleotide prime extension is performed. The result obtained after the single nucleotide primer extension is then quantified for obtaining the amount of methylated DNA [55]. More recently, ion pair reverse phase HPLC and Matrix assisted laser desorption ionisation/time-of-flight methods are used for analysis and calculation of the extended sequences and C:T ratio [55].

### 3.5. Other Epigenetic Methods

Methylated DNA immunoprecipitation (MeDIP) is an antibody-mediated methyl-specific fractionation that can assist in detection of only methylation-based epigenetic modifications [56]. Similarly, high-resolution melting analysis (HRM) based on the calculation of the C: T ratio is used to identify the SNPs (single nucleotide polymorphisms) [57]. To determine the degree of methylation, direct hybridization of CpG island arrays is utilised, either with radioactive or fluorescent probes [58,59]. Melting curve analysis, or Mc-MSP in short, is based on the analysis of the melt curve of the amplified product [60]. Herein the bisulfite treated DNA are amplified, followed by annealing with two types of primers for each methylated and unmethylated DNA. Sergey Kurdyukov and Martyn Bullock have recently elaborated the methods for epigenetics and strategies for choosing the correct method [61].

## 4. Epigenetics in COVID: General

**Figure 3:**
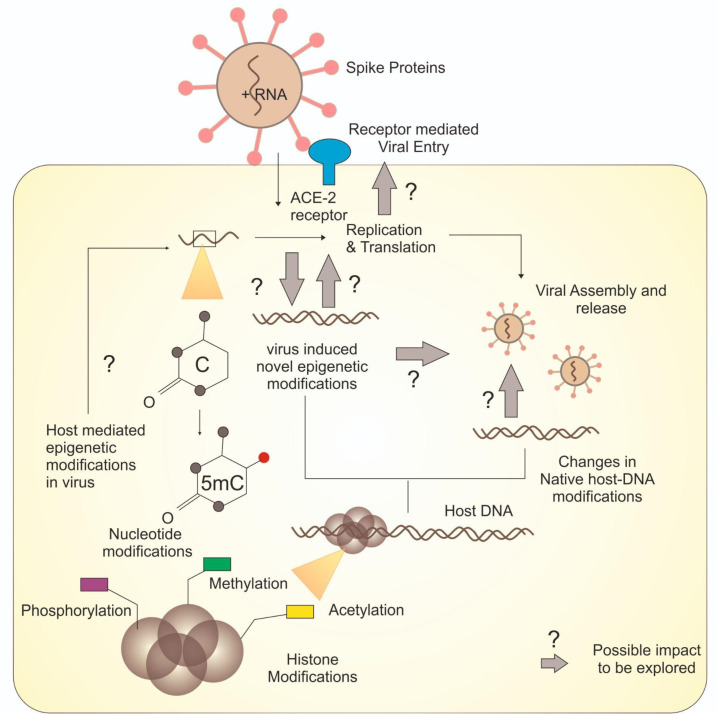
Overview of various epigenetic modifications and unresolved molecular cascades.

Unlike many other diseases, recently emerged COVID-19 infection is also known to be affected by the environment at the genetic level. The epigenetic manifestations of COVID-19 have recently begun to be unraveled and appeared as important prospects for the development of new drugs. Epigenetic changes are associated with almost all types of disease and play pivotal roles in shaping the host-bias of the pathogen and also infectivity and pathology [62]. The role of epigenetics in cancer, imprinting disorders, obesity, other lifestyle disorders and even viral infections has been investigated in the past. Epigenetic mechanisms clearly appear as an essential component of the pathophysiology of SARS-CoV-2, which is crucial in determining the fatality rate and severity of the sickness [63,64]. We have known for several decades that almost all viruses use epigenetic regulation and their life cycle in hosts is greatly affected by environmental regulation, in particular the modification at CpG islands which are known to impact enterocytosis and syncytium formation. The aforesaid two features are crucial for the coronavirus’s entry and maintenance in general [63,65]. The fundamental molecular processes that regulate coronavirus pathogenesis under the control of epigenetics are extremely complex and heavily reliant on host–virus interactions. Although with a few differences between DNA or RNA as genetic material, almost all viruses use host epigenetic reprogramming, which is the essential part of their host immune evasion pathways [63]. The epigenetic factors involved in both COVID-19 infection and host–pathogen interactions have recently been studied in various dimensions. Epigenetic changes have been implicated in the pathophysiology and viral infectivity mainly through chromatin remodulation and genome stabilisation. Such changes have also been implicated in the differential infectivity of viruses in tissue specific, host-specific, gender biassed or sexed biased manner [66]. There is a two-way effect of epigenetics on viral infections as that of COVID-19: one is the epigenetic change in the host that modulates the immunoregulatory axis and therefore influences the defense of the pathogen, and the second is the epigenetic modifications within the basic mechanism of replication of the pathogen that affect infection and prevalence [67]. As stated above, the epigenetic modifications predominantly include DNA methylation at chromatin level leading to histone modifications which eventually lead to modelling of chromatin. The main components involved in this process are known to be modifying proteins such as several types of sirtuins and non-coding RNA including sRNA, miRNA or long non-coding RNAs [68]. Histone-modifying enzymes are yet another class of catalysts that facilitate epigenetic processes. More than a hundred enzymes are involved in the histones protein’s epigenetic modification, some of which are well understood. Histone acetyltransferases/deacetylases are among the widely used histone-modifying enzymes. Histone methyltransferases and histone kinases all have direct effects on chromatin constituents [69]. While some other enzymes that work indirectly include DNA methyltransferase enzymes, the active DNA methylation or demethylation process involve (10,11) translocation proteins and thymine DNA glycosylase. The host epigenome can also be altered by viruses of the Coronaviridae family, in contrast to host-mediated viral epigenetic alterations. Some of the studied epigenetic changes induced by the virus include negative impact on the host immune response which eventually helps the viral survival and spread. The study of viral infection using epigenomics is a novel but effective method, and the enzymes causing epigenetic changes are still possible targets for new antiviral medicines. Therefore, the epigenetic modifications of receptor genes mediated the synthesis and facilitated viral entry make an interesting study choice and represent a new less-explored domain of SARS-CoV-2 epigenetics [70]. Figure 3 illustrates some common epigenetic modifications along with some yet to elucidate molecular cascades.

### 4.1. Viral Entry and Cell Fusion as Potential Epigenetic Targets

The epigenetic mechanisms that regulate COVID-19 have been studied by Sarantis et al. and others, who have specifically emphasized the methylation of ACE2 gene (angiotensin converting enzyme-2), where the modification of this gene have been linked with host-tissue bias, age bias and sex-based bias patterns during COVID-19 infection [66]. As the ACE-2 receptor is directly associated with the viral entry in the cells, the changes in ACE-2 expression caused by epigenetic modifications inevitably influence virulence of the virus. DNA methylation and H3K4 methylation at the gene’s promoter region significantly influence the viral expression profile. The Roadmap Epigenomics Project database [71] analysis also showed that the p300 acetyltransferase and H3K27ac are present at ACE2 gene’s promoter region. These are directly associated with ACE-2 epigenetic in various conditions like smoking or other lung-related comorbidities, particularly in COVID-19 infection. Furthermore, TMPRSS2, a transmembrane protease that has been linked to ACE2 and Spike proteins (S) of the virus during infection, is considered as a crucial regulator for viral infection [72]. The S protein, which is in turn activated by the role of the TMPRSS2 cellular membrane serine protease that causes eventual cleavage, could also be considered as a potential epigenetic target for future studies. The primary epigenetic modifications that have been revealed in studies so far include the histone acetyltransferase1 (HAT1), histone deacetylase 2 (HDAC2) and lysine demethylase 5B (KDM5B) mediated changes which usually control H3K4me1, H3K4me3, H3K27Ac modifications and thereby affect the expression of ACE-2 directly implicated with COVID-19 infection and its pathology. A recent study of the relationship between ACE2’s hypomethylation and increased sensitivity to COVID-19 offers essential details about the epigenetic regulation of viral entry [73]. Further investigation is needed into a unique part of the epigenetic pathway suggested by an intriguing association between COVID-19 vulnerability and cancer patients, where DNA methylation at the ACE2 locus is perhaps involved. It should be noted that the enzyme KDM5B has recently gained interest due to its close association with other viral infections (hepatitis) and possibly with SARS-CoV-2 [74,75]. KDM6B is a histone-specific demethylase of H3K27me3, acting as a repressive histone mark. It is known that this enzyme induces the interferon response, resulting in resistance to infection by both DNA and RNA viruses. Highly homologous histone acetyltransferases, cyclic AMP (cAMP) response element-binding protein or CREB-binding protein (CBP), and p300 are some of the well-established markers of active enhancers [76]. Inhibition of p300 HAT activity has been demonstrated to reduce H3K27Ac levels, mainly at the enhancer regions, and is known to be associated with respiratory epithelial tumorigenesis [77]. This evidence raises the possibility that the CREB epigenetic is also associated with COVID-19, which was recently demonstrated in a system biology-based approach [78]. Additional research on methylation pathways, SARS-CoV-2 -infiltrated lung cells, and cell membrane–virus fusion revealed the active involvement of epigenetics in both lung infiltration and cell membrane [73].

### 4.2. Epigenetic Regulation of Pathogen Replication

Epigenetic regulation of pathogen replication involves change within the genome of the virus or the replication machinery of the host. Lu et al. have recently reported the importance of dynamic epigenetic change occurring in RNA post transcriptionally and its association with the host’s immune system. They described the modification of N6-methyladenosine (m6A in short) that can modify viral activity [79]. This alteration is found in numerous other viruses and is not specific to the coronavirus. The m6A expresses pro- and anti-viral activity depending on the viral species and host cell type, making it an important factor for diagnosing and treating viral infection. The aforementioned modification and another modification m6M are known to modulate the viral replication and innate immune response pathways [80]. Furthermore, Kuppers et al. corroborate the finding that the gene expression, replication, and viral reproduction are modulated to a great extent by m6A modifications. Moreover, m6A methylation helps viruses in evading the host immune system [81]. Furthermore, the RNA methylase complex METTL3/14 has been shown to be able to modify more than 50 possible m6A sites in the SARS-CoV-2 RNA genome, including GGACU (T), GGACA, and GGACC, which is likely to allow the acquisition of m6A alterations in SARS-CoV-2 RNA [79,81]. Therefore, it is interesting that the host cell’s m6A epi-transcriptome is crucial for preventing viral infection and can change after viral infection [82,83]. Another interesting aspect about the entire coronavirus family is that they can encode their own methyltransferases, especially for the purpose of host immune evasion [84]. Therefore, further investigation into this biochemical cascade and m6A as a potential target may be successful and result in the identification of new therapeutic targets as well as potential COVID-19 vaccine candidates.

### 4.3. Dietary Intervention and Epigenetics Targets

Diet has a profound role on epigenetic modification and so is the effect and pathology of any diseases. COVID-19 infection also does not evade this hypothesis and its pathology has been associated with dietary interventions. Dietary strategies aimed at preventing COVID-19 have been recently considered by clinicians on a global scale. In fact, most of the mild infections of COVID-19 were reportedly improved by a healthy and balanced diet [85]. Furthermore, several specific nutrients in diet such as vitamins, minerals, polyphenols, flavanol, etc., present in conventional fruit and vegetable rich diet were reported to be advantageous in early recovery from COVID. It is well established that body immunity is an essential factor to minimize the seriousness of COVID-19, likely by modulating the immune response as has been previously documented for multiple other diseases including cancer, HIV, and other pathogen- or lifestyle-induced disorders. Numerous nutrients that individuals ingest and are utilised in conventional therapy, including vitamin C, probiotics, and vitamin D, have been demonstrated to include elements with anti-inflammatory, antibacterial, and antiviral activities [86]. The role of vitamin D, selenium, and probiotics in mitigating or enhancing conventional therapies has been recently proposed and documented [87,88]. In addition, vitamin C, a safe and necessary supplement, was found by Harry Hemila et al. to dramatically shorten the duration of stay in the intensive care unit (ICU) by an average of 8% in their randomised placebo-controlled trials, and also reduce the time that ICU patients with respiratory illnesses spend on mechanical ventilation [89]. Whether or not these dietary interventions are associated with enhancing the COVID-19 severity via epigenetic modifications is not well-documented, and therefore requires further explorations. Some studies, however, provide preliminary evidence to support the notion in the context of other pathological conditions, recently reviewed. Numerous studies have suggested that phenolic substances can influence gene expression by controlling epigenetic processes such as DNA methylation, histone modification, or miRNA expression [90]. Generally, various phenolic compounds can activate HDACs (e.g., fisetin, genistein) or inhibit (e.g., EGCG, curcumin) HATs. Based on various studies, it is likely that diet-based disparities and COVID-19 recovery and pathophysiological differences are possibly linked to polyphenolic targets or steroidal targets which need to be extensively evaluated for epigenetic modification to clearly understand the phenomenon.

### 4.4. Epigenetics of COVID Immunity

The most perplexing question is the explanation for the wide diversity of symptoms and also the immune response of COVID-19, which makes it more challenging to understand and also much more likely to be associated with epigenetics [91]. The unusual epidemiological data from African children showed that host–pathogen interaction is closely coupled to epigenetic regulation of cognitive compartments belonging to innate immunity [88]. Along with the epigenetic regulation of the COVID-immunity, the effectiveness of vaccines is also influenced by epigenetic rules. It has been proposed in the past that a vaccine’s capacity to build immunological memory by innate immune cells, instead of just eliciting a response from lymphocytes, may be connected to how well it protects against various infections. This improved the antimicrobial function of innate immune cells found in live vaccines, enabling a quicker response when exposed to reinfections. This prompted researchers to find solid support for the influence of long-lasting epigenetic modifications [92]. A positive-sense RNA virus called severe acute respiratory syndrome coronavirus-2 is the cause of the ongoing COVID-19 pandemic. The entrance of the host cell is controlled by ACE2R methylation at three CpG sites (cg04013915, cg08559914, and cg03536816). By regulating the SIRT1 and KDM5B activities it controls the expression of ACE2. Additionally, it modifies the histone modifications H3K27me3 and H3K4me3 to control Type I and III IFN responses. Bromodomain and protein E-containing SARS-CoV-2 protein imitates bromodomain histones and thwart host immune response. The 2’-O MTases play a critical function in immune evasion through an Hsp90-mediated epigenetic process to hijack the infected cells by mimicking the host’s cap1 structure. Although the crucial epigenetic events linked to SARS-CoV-2 immune evasion were emphasized in the analysis, the precise mechanism is still unclear. A more detailed aspect has been recently reviewed by Bimal Jit et al. [91]. Furthermore, a few more recent reports have provided concrete evidence of how SARS-CoV-2 infections alter the host genes’ epigenetic state. Some of the potential targets identified in these studies included DTX3L, HDAC7, HDGF, PRDM1, PRMT1, and TRIM16 were upregulated, whereas FOXO1, HELLS, PADI3, and PPARGC1A were downregulated [93]. There are several other aspects of alteration in the immune response of the host by SARS-CoV-2 via epigenetic alterations. One of them is the alteration of antigen presentation and Interferon response, while others include mimicry of histones for modulation of gene expression and immune evasion. Significant interferon-stimulated gene expression delay was caused by SARS-CoV-2. SARS-CoV-2 may control Type I and III IFN response by modifying the histone modifications H3K27me3 and H3K4me3. Acetylation and deacetylation of histones cause the inflammatory response associated with COVID-19 that is mediated by monocytes and macrophages. Bromodomain and protein E-containing SARS-CoV-2 protein imitates bromodomain histones and thwart host immune response. Additionally, it is hypothesized that SARS-CoV-2 may control NF-B signaling by recruiting p65 to the chromatin [91]. There is, however, a dearth of relevant material in this area and it needs further exploration.

### 4.5. Epigenetic and Old-Age COVID Mortalities

The epidemiology of mortality in COVID-19 clearly suggests a proportionately larger influence on the elderly population. As epigenetic factors are dynamic and variable temporally and remain variable with age. It seems obvious that age-related increases in morbidity and death may also be linked to the host’s epigenetic changes [94]. Numerous investigations and clinical data sets made it abundantly clear that older people contributed to both a higher proportion of infected cases and a higher risk of mortality [95,96]. The entire ageing process can be explained as the result of DNA methylation, which eventually regulates the biological clock in many cells. Furthermore, aging-related alterations have a deleterious impact on the immune system, immunological memory loss, and innate, adaptive, and antiviral defenses. Various studies have recently started to explore the predilection with SARS-CoV-2 mediated epigenetic changes, and it has been observed that the virus may speed up the immune system’s aging process by a gene silencing method that produces the MHC molecules (major histocompatibility complexes), inhibiting host antigen presentation by changing DNA methylation and antagonising host antigen presentation [97]. Viral Nsp5 protein interacts with HDAC1 and acts as a potential epigenetic regulator. Moreover, it is now becoming clearer that the collective effect of epigenetic insults can delay interferon response genes and influence the host defense and therefore comorbidities [97]. Furthermore, age-related SARS-CoV-2 pathogenesis is linked to the ACE receptor, the virus’ primary point of entrance into the host cell. ACE2, like most other gene expression systems, is generally regulated in the body during the transcriptional and translational process and is known to be controlled by methylation. Studies on both human subjects and animal models have demonstrated that methylation at one of seven CpGs in the ACE2 promoter declines with ageing. Studies in mice and rats have also shown that ACE2 expression declines with ageing and is linked to aortic fibrosis and inflammation [98]. Contrary to results, Franzen et al. investigated several DNA methylation datasets of blood samples with epigenetic aging signatures and carried out targeted bisulfite amplicon sequencing. They came to the conclusion that overall, epigenetic clocks were not significantly accelerated in COVID-19 patients and disregarded the unreliability of these markers to anticipate an increased risk for serious COVID-19 infection in older patients [99].

### 4.6. Epigenetic Significance of COVID-19

It is evident from the aforementioned completed or ongoing studies that epigenetic modifications have a profound impact in the pathology and progression of COVID-19 infection in humans. Moreover, the native epigenetic modifications in humans have also been established by research as potential disease predisposition markers. However, it is not yet elucidated how these modifications can be modulated by potential chemicals or metabolites in a controlled fashion so that they can be developed as potential prophylactics or therapeutics. A deeper understanding of epigenetic regulations during COVID-19 infections would not only provide us with an opportunity to better understand the pathology of the disease, but also allow us to modulate the process at genetic level, which remains at the origins of any diseases. Furthermore, a better understanding of cross-talk across the epigenetic modifications of humans and that of viral-DNA would also enable us to develop better genomic-level therapeutic and precision medicine for high vulnerability groups such as diabetic or cardiovascular patients infected by SARS-CoV-2.

## 5. Conclusions

The stabilization of the genome and preservation of cellular homeostasis are significantly affected by epigenetic alterations that control the shape of chromatin, and these changes have been linked to the pathophysiology of viral infection. Provided the scale and quantum of the global effect of SARS-CoV-2 infection, the mammoth task of exploring the complete biology of infection is ongoing. Provided with a high degree of variability in pathophysiology and bias toward age, sex, and comorbidities, the COVID-19 infection is reportedly regulated by epigenetic modifications both of virus and host. Several targets of epigenetic modification in the virus, as well as the host, have been identified including m6A, ACE2 modifications, etc., which have been linked to various conditions in infected hosts and linked to their pathology. However, what remains to be clearly understood is how these modifications are regulated and what causes their onset; subsequently, how these modifications would result in the age-related, sex-related, and comorbidities-related bias in COVID-19 conditions. Moreover, the detailed exploration of the targets of epigenetic modification shall also provide a possible lead for the development of bias-independent and more effective therapeutic interventions.
